# EZH2 enhances the differentiation of fibroblasts into myofibroblasts in idiopathic pulmonary fibrosis

**DOI:** 10.14814/phy2.12915

**Published:** 2016-08-31

**Authors:** Xiao Xiao, Lakmini K. Senavirathna, Xuxu Gou, Chaoqun Huang, Yurong Liang, Lin Liu

**Affiliations:** ^1^ Oklahoma Center for Respiratory and Infectious Diseases Oklahoma State University Stillwater Oklahoma; ^2^ Department of Physiological Sciences Lungberg‐Kienlen Lung Biology and Toxicology Laboratory Stillwater Oklahoma

**Keywords:** EZH2, fibroblasts, IPF, myofibroblasts, Smad2/3

## Abstract

The accumulation of fibroblasts/myofibroblasts in fibrotic foci is one of the characteristics of idiopathic pulmonary fibrosis (IPF). Enhancer of zeste homolog 2 (EZH2) is the catalytic component of a multiprotein complex, polycomb repressive complex 2, which is involved in the trimethylation of histone H3 at lysine 27. In this study, we investigated the role and mechanisms of EZH2 in the differentiation of fibroblasts into myofibroblasts. We found that EZH2 was upregulated in the lungs of patients with IPF and in mice with bleomycin‐induced lung fibrosis. The upregulation of EZH2 occurred in myofibroblasts. The inhibition of EZH2 by its inhibitor 3‐deazaneplanocin A (DZNep) or an shRNA reduced the TGF‐*β*1‐induced differentiation of human lung fibroblasts into myofibroblasts, as demonstrated by the expression of the myofibroblast markers *α*‐smooth muscle actin and fibronectin, and contractility. DZNep inhibited Smad2/3 nuclear translocation without affecting Smad2/3 phosphorylation. DZNep treatment attenuated bleomycin‐induced pulmonary fibrosis in mice. We conclude that EZH2 induces the differentiation of fibroblasts to myofibroblasts by enhancing Smad2/3 nuclear translocation.

## Introduction

Idiopathic pulmonary fibrosis (IPF) is a specific form of chronic, progressive fibrosing interstitial pneumonia and is associated with the histopathologic and radiologic characteristics of usual interstitial pneumonia (UIP) (Raghu et al. 2011; Visscher and Myers 2006). IPF is characterized by the deterioration of respiratory functions due to fibrosis of the lung interstitium. A majority of deaths (77%) from IPF are due to respiratory‐related causes, including the progression of IPF, acute exacerbation, acute lung injury, pneumonia, and cor pulmonale. The causes of other deaths (23%) include cardiac causes, sepsis, cancer, and so on (Ley et al. 2011). The potential risk factors are cigarette smoking (Baumgartner et al. 1997; Raghu et al. 2011), aging (Faner et al. 2012), environmental exposures to metal (brass, lead, and steel) and wood (pine) dusts (Hubbard et al. 1996; Miyake et al. 2005), chronic viral infection (Epstein–Barr virus and hepatitis C) (Egan et al. 1995; Irving et al. 1993; Ueda et al. 1992), gastroesophageal reflux (GER) (Gribbin et al. 2009; Raghu et al. 2006), and diabetes mellitus (Gribbin et al. 2009). Several studies have suggested that patients may be genetically predisposed to IPF. Mutations in *sftpc* and *spfta2* genes (Chibbar et al. 2004; Lawson et al. 2011; Maitra et al. 2010; Nogee et al. 2002; Thomas et al. 2002; van Moorsel et al. 2010; Wang et al. 2009) and an increase in common polymorphisms of the *muc5b* gene (Peljto et al. 2012; Seibold et al. 2011) are associated with IPF.

The current view for the pathogenesis of IPF is that repetitive epithelial injuries cause abnormal activation of the epithelial cells and aberrant wound healing, leading to the formation of the fibroblast/myofibroblast foci. The activated fibroblasts can differentiate into contractile and secretory myofibroblasts, which cause excessive contraction and extracellular matrix (ECM) deposition. The differentiated myofibroblasts secrete angiotensinogen and hydrogen peroxide, which induce alveolar epithelial cell apoptosis. They also produce matrix metalloproteinases, which disrupt the basement membrane (Waghray et al. 2005; Wang et al. 1999). The fibroblasts and myofibroblasts in IPF are more resistant to apoptosis and produce persistent foci in the lung tissue (Kulasekaran et al. 2009).

Enhancer of zeste homolog 2 (EZH2) is the catalytic component of a multiprotein complex, polycomb repressive complex 2 (PRC2). PRC2 catalyzes the trimethylation of histone H3 at lysine 27 (H3K27me3). EZH2 contains a SET domain, which provides the methyltransferase active site, although EZH2 alone exhibits no intrinsic enzymatic activity. To attain catalytic activity, it must be complexed with at least two proteins, embryonic ectoderm development (EED) and suppressor of zeste 12 (SUZ12) (Cao and Zhang 2004; Pasini et al. 2004; Rea et al. 2000). These three proteins combine with the histone‐binding proteins, retinoblastoma‐binding protein 4 (RBBP4) and RBBP7, to form the core components of PRC2 (Chase and Cross 2011). In addition to interacting with the PRC2 components, EZH2 also interacts with other proteins, including the transcription factor Yin Yang 1 (YY1) (Caretti et al. 2004; Wilkinson et al. 2006) and the nuclear inhibitor of protein Ser/Thr phosphate‐1 (NPP1) (Van et al. 2010).

Overexpression of EZH2 has been associated with several cancers, including prostate cancer, breast cancer, bladder cancer, and lung cancer (Simon and Lange 2008; Yang and Yu 2013). Cigarette smoke induces EZH2‐mediated repression of the Wnt signaling inhibitor Dickkopf‐1 in lung cancer cells (Hussain et al. 2009). EZH2 is also the target of miR‐101, and the genomic loss of miR‐101 in cancer leads to overexpression of EZH2, resulting in cancer progression (Cao et al. 2010; Varambally et al. 2008). However, the role of EZH2 in IPF is unknown. One study has shown a role of EZH2‐mediated hypermethylation in the epigenetic silencing of cyclooxygenase 2 (COX‐2), which reduces the production of the antifibrotic protein prostaglandin E2 (Cao et al. 2010).

IPF and cancer are thought to share similar characteristics, such as genetic alternations, epigenetic alterations, myofibroblast invasion, and abnormal activation of specific signaling pathways (Vancheri 2013; Vancheri et al. 2010). This implies that some of the mechanisms used in cancer may also be involved in IPF. The objectives of this study are to determine the EZH2 expression in human patients with IPF and in bleomycin‐induced murine pulmonary fibrosis, effects of EZH2 inhibition on the differentiation of fibroblasts to myofibroblasts, how EZH2 affects the TGF‐*β* signaling pathway, and the effects of EZH2 inhibition on pulmonary fibrosis in an in vivo mouse model. Our study discovers a novel role for EZH2 in IPF and provides a possible therapeutic target for IPF treatment.

## Materials and Methods

### Human lung tissue

Twenty‐eight lung tissue samples from IPF patients were obtained from the Lung Tissue Research Consortium (LTRC). The tissues were divided into three groups according to the predicted probronchodilator forced vital capacity (FVC) of the patients: >80% (*n* = 8), 50–80% (*n* = 10), and <50% (*n* = 10). The lung tissues were stored in RNAlater solution at −80°C until further use.

### Culture of the human LL29 cells

Human lung fibroblasts (LL29, AnHa), which were isolated from a 26‐year‐old Caucasian female, were purchased from the American Type Culture Collection (ATCC, Manassas, VA) and cultured in F‐12K medium (Kaighn's modification of Ham's F‐12 medium, ATCC) supplemented with 1% penicillin/streptomycin and 10% heat‐inactivated fetal bovine serum (FBS). The cells (1.6 × 10^5^ per well) were cultured in complete F12‐K culture media in 12‐well plates. After reaching 70% confluence, the cells were treated with 4 *μ*M 3‐deazaneplanocin A hydrochloride (DZNep; Sigma‐Aldrich, St. Louis, MO) for 24 h, followed by stimulation with 5 ng/mL of recombinant human TGF‐*β*1 (R&D Systems, Minneapolis, MN) for various amounts of time. The cells were then washed with ice‐cold phosphate‐buffered saline (PBS) and collected with either Tri Reagent (Molecular Research Center, Cincinnati, OH) to isolate the RNA or the M‐PER Mammalian protein extraction reagent (Thermo Fisher Scientific, Grand Island, NY) plus Halt protease and/or phosphatase inhibitor cocktail to extract the proteins.

### Real‐time PCR

The total RNA was isolated from the cultured LL29 cells or the human or animal lung tissues using TRI Reagent, according to the manufacturer's instructions. The RNA concentrations and quality were determined using a NanoDrop ND‐1000 spectrophotometer (NanoDrop Tech., Rockland, DE). The A260/A280 and A260/A230 of the RNA samples were >1.8 and 1.7, respectively. The RNA was treated with TURBO DNase (Ambion, Austin, TX) to remove the genomic DNA contamination. One microgram of RNA was reverse‐transcribed into cDNAs using M‐MLV reverse transcriptase, random primers, and oligo‐dT primers (Promega, Madison, WI). The primers were designed using Primer Express software (Applied Biosystems) and are listed in Table 1. Real‐time PCR was performed on a 7900HT Fast Real‐Time PCR System (Applied Biosystems) using SYBR Green I–Low ROX (AnaSpec, Fremont, CA). The thermal conditions were 95°C for 10 min, followed by 40 cycles at 95°C for 15 sec, and 60°C for 60 sec. The mRNA levels of each gene were calculated using the comparative CT method.

**Table 1:** Primers used for real‐time PCR

Human‐EZH2‐FW	TCCTACATCCTTTTCATGCAACAC
Human‐EZH2‐RE	CCCTCCAAATGCTGGTAACAC
Mouse‐EZH2‐FW	TGTGACCCTGACCTCTGTCTCA
Mouse‐EZH2‐RE	AGACGGTGCCAGCAGTAAGTG
Human‐18S‐FW	CGTTGATTAAGTCCCTGCCCTT
Human‐18S‐RE	TCAAGTTCGACCGTCTTCTCAG
Mouse‐18S‐FW	ATTGCTCAATCTCGGGTGGCTG
Mouse‐18S‐RE	CGTTCTTAGTTGGTGGAGCGATTTG
Human‐GAPDH‐FW	GAAGGTGAAGGTCGGAGTCAAC
Human‐GAPDH‐RE	CATGGGTGGAATCATATTGGAA
Human‐ACTB‐FW	GGCACCACACCTTCTACAATGA
Human‐ACTB‐RE	ACAGCCTGGATAGCAACGTACA
Mouse‐GAPDH‐FW	CTCGTCCCGTAGACAAAATGGT
Mouse‐GAPDH‐RE	TGATGGCAACAATCTCCACTTT
Mouse‐ACTB‐FW	GGCCAACCGTGAAAAGATGA
Mouse‐ACTB‐RE	TCCATCACAATGCCTGTGGTA
Human‐ *α*SMA‐FW	GTGTTGCCCCTGAAGAGCAT
Human‐ *α*SMA‐RE	CGCCTGGATAGCCACATACAT
Mouse‐ *α*SMA‐FW	ATCCGATAGAACACGGCATCA
Mouse‐ *α*SMA‐RE	CAGCAGTGTCGGATGCTCTTC
Human‐fibronectin‐FW	CCTGCATCTGAGTACACCGTATC
Human‐fibronectin‐RE	GGTCTCAGTCACCTCGGTGTT
Mouse‐COL1A1‐FW	ACGCATGGCCAAGAAGACAT
Mouse‐COL1A1‐RE	TTGTGGCAGATACAGATCAAGCA
Mouse‐COL3A1‐FW	CACCCTTCTTCATCCCACTCTT
Mouse‐COL3A1‐RE	TGACATGGTTCTGGCTTCCA

The primers were designed using Primer Express software (Applied Biosystems). Length of primers is between 18 and 30 nt with a GC content between 40% and 60%. FW, forward; RE, reverse.

### Western blotting

10–20 *μ*g of the proteins were separated on SDS‐PAGE gels and transferred to a nitrocellulose membrane. The membranes were blocked with 10% nonfat milk for 60 min at room temperature, and then incubated with primary antibodies overnight at 4°C. The resources and dilutions of the primary antibodies are rabbit anti‐EZH2 monoclonal antibody (5246, Cell Signaling, 1:500), mouse anti‐ACTA2 (*α*‐SMA) monoclonal antibody (SAB1403519, SIGMA, 1:10,000), rabbit anti‐GAPDH polyclonal antibody (G9545, SIGMA, 1:10,000), rabbit anti‐p‐Smad2/3 monoclonal antibody (8828, Cell Signaling; 1:500), rabbit anti‐Smad2/3 monoclonal antibody (8685, Cell Signaling; 1:500), rabbit anti‐p‐Smad2 monoclonal antibody (3108, Cell Signaling; 1:500), rabbit anti‐p‐Smad3 monoclonal antibody (9520, Cell Signaling; 1:500), rabbit anti‐ACTB (*β*‐actin) polyclonal antibody (A2066, SIGMA, 1:10,000), and rabbit anti‐Lamin B1 monoclonal antibody (12586, Cell Signaling, 1:500). The membrane was washed using Tris‐buffered saline with Tween 20 (TTBS) and incubated with goat anti‐mouse or goat anti‐rabbit secondary antibodies (115‐035‐003 or 111‐035‐003, Jackson ImmunoResearch, West Grove, PA, 1:10000) for 1 h. The blots were then developed with enhanced chemiluminescence (ECL) reagents (PIERCE, Rockford, IL) and imaged with an Amersham Imager 600 (GE Healthcare Bio‐Sciences, Pittsburgh, PA).

### Gel contraction assay

The cells were mixed with collagen type 1 solution (BD Biosciences, San Jose, CA) with a final concentration of 1 × 10^5^ cells/mL and 1 mg/mL of collagen. 15 *μ*Lof 0.5 N NaOH was added to 1 mL of the mixture. An aliquot of 500 *μ*L of the mixture was immediately distributed to each well of 12‐well plates, and the plates were incubated in a cell culture incubator for 30 min to polymerize the gel. The polymerized gel was detached from the well wall and bottom using a 200‐*μ*L pipette tip, and 500 *μ*L of complete growth medium with or without DZNep and/or TGF‐*β*1 were added to each well. Photos of each well were taken after 48 h. Using ImageJ software (http://imagej.net/ImageJ), the surface areas of the gel and the bottom of the well were measured. The gel contraction activity was expressed as a ratio of the surface areas of the gel to the bottom of the well.

### Extraction of the nuclear and cytoplasmic fractions

The nuclear and cytoplasmic fractions were separated using the NE‐PER nuclear and cytoplasmic extraction reagents (Thermo Fisher). The LL29 cells were harvested using trypsin–EDTA and then centrifuged at 500 *g* for 5 min. The cell pellet was washed with PBS and centrifuged at 500 *g* for 3 min. The supernatant was removed, leaving the cell pellet as dry as possible. 100 *μ*L of the CER I reagent with proteinase inhibitors was added to the tube. The cells were fully suspended by vigorous vortexing and then incubated on ice for 10 min. CER II (5.5 *μ*L) was then added. The tubes were vortexed for 5 sec and centrifuged at 20,000 *g* for 5 min. The supernatant (cytoplasmic extract) was transferred to a new tube and stored at −80°C. The remaining pellet was suspended in 50 *μ*L of NER reagent with proteinase inhibitors. The tubes were vortexed vigorously for 15 min and placed on ice; the tubes were vortexed for 15 sec every 10 min for a total of 40 min. The tubes were then centrifuged at 20,000 *g* for 10 min, and the supernatant (nuclear extract) was transferred to a new tube and stored at −80°C for the western blotting analysis.

### Immunohistochemistry

The lung tissue sections were deparaffinized twice in xylene for 1 min and were successively hydrated in 100%, 95%, and 70% alcohol and deionized water. The endogenous peroxidase activity was quenched by incubating the sections with 0.3% H_2_O_2_ in water for 30 min at room temperature. The slides were blocked with 1.5% goat serum in PBS for 20 min at room temperature. The slides were then incubated with rabbit anti‐EZH2 monoclonal antibodies (1:200 dilution, Cell Signaling Technology, Danvers, MA) in 1.5% goat serum in PBS for 60 min at room temperature. After washing with PBS for 5 min, the slides were incubated with goat anti‐rabbit biotinylated secondary antibodies (1:200 dilution) for 30 min at room temperature. Then, the slides were incubated with the ABC staining reagent (Vectastain, Burlingame, CA) for 30 min. After the slides were washed in PBS for 5 min, they were incubated in a peroxidase substrate to develop the desired color.

For EZH2 and *α*‐SMA double staining, the procedure for EZH2 ABC staining was the same as described above. Following incubation with the anti‐EZH2 antibodies, the tissue sections were incubated with anti‐ *α*‐SMA antibodies (1:200 dilution) together with the anti‐EZH2 antibodies overnight at 4°C. ABC staining was performed to detect EZH2, followed by incubation with an AlexaFluor 488‐conjugated rabbit IgG fluorescent secondary antibody (1:3000, Life Technologies, Grand Island, NY) to detect *α*‐SMA. The bright field and fluorescence images were captured in the same field.

### Isolation of the primary mouse fibroblasts/myofibroblasts

The fibroblasts and myofibroblasts were isolated from 10‐week‐old female C57BL/6 mice with or without a bleomycin challenge as described previously (Bruce et al. 1999; Homolya et al. 1999). The lungs were first trimmed to remove the major airways and rinsed with PBS. The lung tissue then was transferred into 100‐mm cell culture dishes and minced into 1–2 mm^3^ pieces. The minced lung tissue was incubated with 5 mL of 0.25% trypsin–EDTA (Thermo Fisher Scientific) in a cell culture incubator at 37°C. At each of three 10‐min intervals, the dishes were gently shaken by hand. After 30 min, the cells were centrifuged at 250 *g* for 10 min at 4°C. The pellet was suspended in 10 mL of Dulbecco's modified Eagle's medium (DMEM) supplemented with 10% FBS, and transferred into 100 mm dishes. After 1 week of culture in the 37°C incubator, the nonadherent cells were removed and the adherent cells were trypsinized and passed through sterile 70‐*μ*m cell strainers (Thermo Fisher Scientific). The filtered fibroblasts/myofibroblasts were cultured in DMEM with 10% FBS until they reached 100% confluency and were then stored in liquid nitrogen until further use.

### Construction of the shRNA vector

We used BLOCK‐iT RNAi Designer, an online program from Thermofisher.com, to design the oligonucleotides for the EZH2 shRNAs. The highest ranked target sequence, GCAGCTTTCTGTTCAACTTGA, was chosen. For the forward (FW) oligo, GATCC, a sequence for *BamH*I restriction enzyme was added to the 5′ end of the target sequence. TTCAAGAGA, a loop sequence, was added to the 3′ end, followed by antisense sequence. TTTTTG was added to the 3′ end of the oligo. The final sequence was GATCCGCAGCTTTCTGTTCAACTTGATTCAAGAGATCAAGTTGAACAGAAAGCTGTTTTTG. For the reverse (RE) oligo, AATTCAAAA, a sequence for the EcoR1 restriction enzyme, was added to the 5′ end of the target sequence. A loop sequence, TCTCTTGAA, was added between the target sequence and the antisense sequence. Another G was added to the 3′ end. The final sequence was AATTCAAAAAGCAGCTTTCTGTTCAACTTGATCTCTTGAATCAAGTTGAACAGAAAGCTGG. The FW and RE oligos were annealed in annealing buffer (Promega). The pGreenPuro vector (System Biosciences, Mountain View, CA) was first double‐digested using BamH1 and EcoR1 restriction enzymes. The annealed oligo was then ligated to the digested pGreenPuro vector using T4 ligase (Promega). The plasmids were transformed and amplified in competent cells (*E. coli* ST3). The final plasmids were isolated using the QIAprep Spin Miniprep Kit (Qiagen, Valencia, CA). The sequence of the insert was confirmed by sequencing. The shRNA lentiviruses were produced in HEK293T cells and their titers were determined as described previously (Xiao et al. 2015).

### Mouse model of pulmonary fibrosis

The 10‐week‐old female C57BL/6 mice (The Jackson Laboratory, Bar Harbor, ME) were housed in the Animal Resources at Center for Veterinary Health Sciences, Oklahoma State University. The animal procedures were approved by the Institutional Animal Care and Use Committee (IACUC) at the Oklahoma State University. The mice were randomly divided into four groups and anesthetized using an intraperitoneal injection of a ketamine and xylazine mixture. The mice were then challenged with 50 *μ*L of PBS or 1 unit/kg of bleomycin (Sigma‐Aldrich) in 50 *μ*L of PBS through an intratracheal route. The EZH2 inhibitor DZNep (2 mg/kg in 300 *μ*L PBS) was administered daily through an intraperitoneal route beginning 1 day before bleomycin instillation until day 17. The lung tissues were collected on day 17 after the bleomycin challenge. The right lung was fixed with 4% formaldehyde for H&E staining and immunostaining. The left lung was minced and then ground into a powder in liquid nitrogen; half of the sample was homogenized using Tri Reagent for real‐time PCR and another half was homogenized using the M‐PER protein lysis buffer for western blotting. The severity of the pulmonary fibrosis was evaluated using Ashcroft scores as described previously (Ashcroft et al. 1988). The evaluator was blinded to the group.

### Statistical analysis

The statistical analyses were performed using GraphPad by Student's *t*‐test for two independent groups and one‐way ANOVA, followed by Tukey's post hoc test, or two‐way ANOVA, followed by Bonferroni's post hoc test for multiple comparisons. *P* < 0.05 was considered significant.

## Results

### EZH2 is upregulated in the lungs of patients with IPF and in mice with bleomycin‐induced lung fibrosis

**Figure 1:**
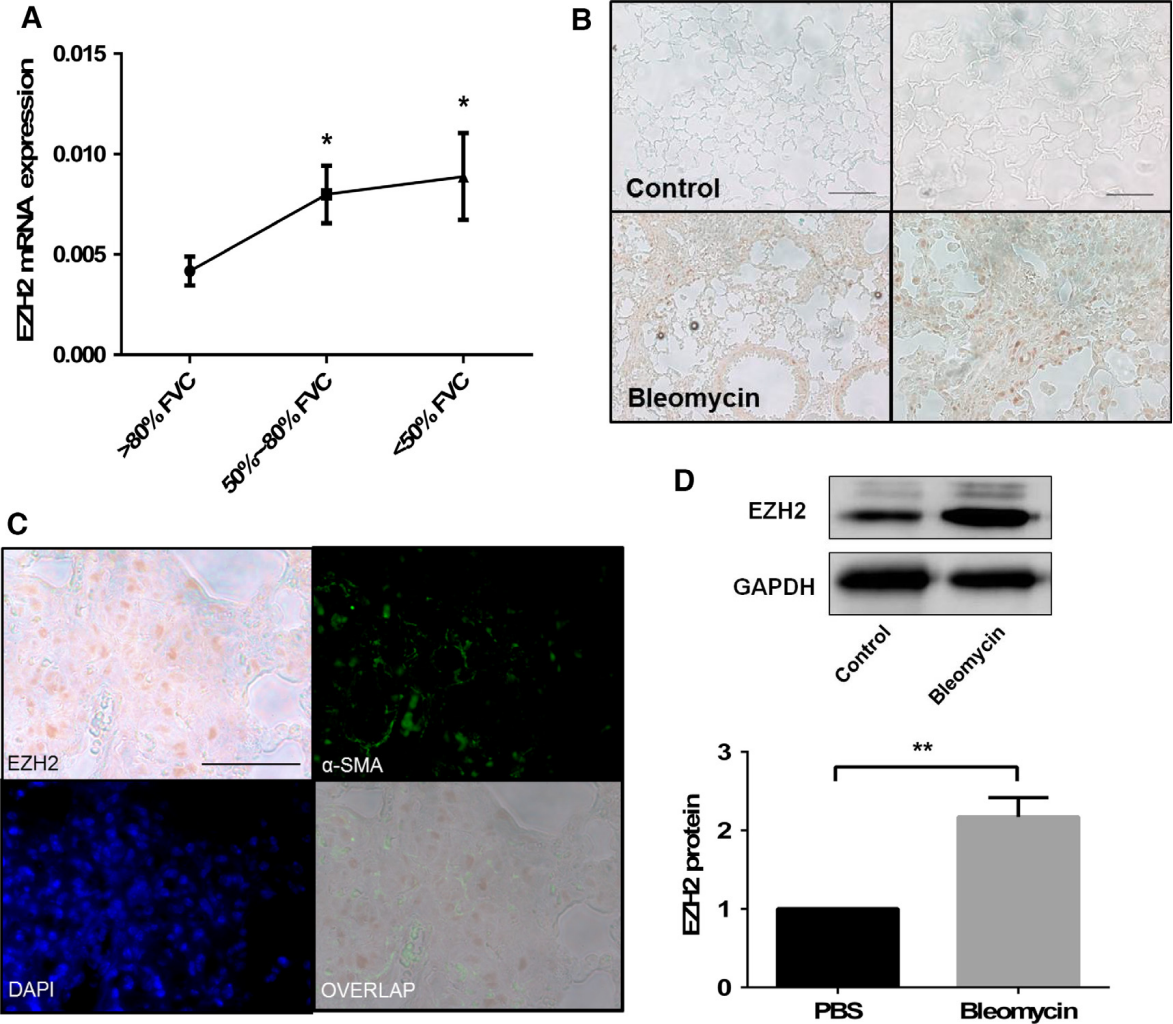
EZH2 expression in the lungs of patients with IPF and in bleomycin‐challenged mice. (A) The lung tissues from patients with IPF were grouped according to their forced vital capacity (FVC): mild, >80% (8 patients), medium, 50–80% (10 patients), and severe, <50% (10 patients). The expression of the EZH2 mRNA was analyzed by real‐time PCR and normalized to the *β*‐actin levels. The results are shown as the mean ± SE. *P < 0.05 versus the >80% FVC group (Student's *t*‐test). (B) The lung tissue sections from the PBS‐ (control) and bleomycin‐challenged mice were stained with anti‐EZH2 antibodies and ABC. EZH2 is shown in brown color. Scale bars: left 100 *μ*m and right 50 *μ*m. (C) The lung tissue sections were double‐labeled with ABC staining using anti‐EZH2 antibodies and immunofluorescence staining using anti‐ *α*‐SMA antibodies. The nuclei were stained with DAPI. An overlay of the EZH2 and *α*‐SMA staining showed that the two proteins were colocalized. Scale bar: 50 *μ*m. (D) The primary fibroblasts/myofibroblasts were isolated from the PBS control and bleomycin‐challenged mice. The EZH2 protein levels were determined by western blotting. The western blot results were quantified using ImageJ software and normalized to the GADPH levels. The results are shown as the mean ± SE from three independent experiments, in which each experiment used different generations of primary fibroblasts/myofibroblasts from four animals (two control and two bleomycin‐challenged mice). **P < 0.01 (Student's *t*‐test).IPF, idiopathic pulmonary fibrosis; PBS, phosphate‐buffered saline.

To determine the potential roles of EZH2 in IPF, we first measured the expression of the EZH2 mRNA in the lung tissues from patients with IPF. The tissues were divided into three groups according to the disease severity and the patients’ pulmonary function, which was determined by the predicted FVC: mild group, >80%; medium group, 50–80%; and severe group, <50%. We found that the expression of the EZH2 mRNA was increased and positively correlated with the severity of the disease (Fig. 1A).

We also examined the EZH2 expression in the lung tissues of a bleomycin‐induced murine lung fibrosis model. Immunohistochemical analysis using EZH2 antibodies showed a strong staining in the bleomycin group, whereas the signals were virtually undetectable in the control group (Fig. 1B), suggesting that the EZH2 protein is also upregulated in bleomycin‐induced lung fibrosis.

To determine whether the increased levels of the EZH2 protein were present in myofibroblasts, we performed double‐labeling with antibodies against EZH2 and *α*‐SMA, a cellular marker for myofibroblasts. We used ABC staining to detect the EZH2 protein and immunofluorescence staining to detect the *α*‐SMA protein in the same section. Positive signals for EZH2 (brown) and *α*‐SMA (green) were observed in the fibrotic foci from the lungs of the bleomycin‐challenged mice. When the images from the staining with each antibody were overlaid, the EZH2 and *α*‐SMA signals were colocalized in the same cells (Fig. 1C). To further confirm this finding, we isolated primary fibroblasts/myofibroblasts from the lungs of mice challenged with bleomycin or PBS (control). A western blotting analysis revealed that the EZH2 protein was increased in the fibroblasts/myofibroblasts from the bleomycin‐challenged mice compared to those from the control mice (Fig. 1D).

### EZH2 is required for the TGF‐***β***1‐induced differentiation of fibroblasts to myofibroblasts

**Figure 2:**
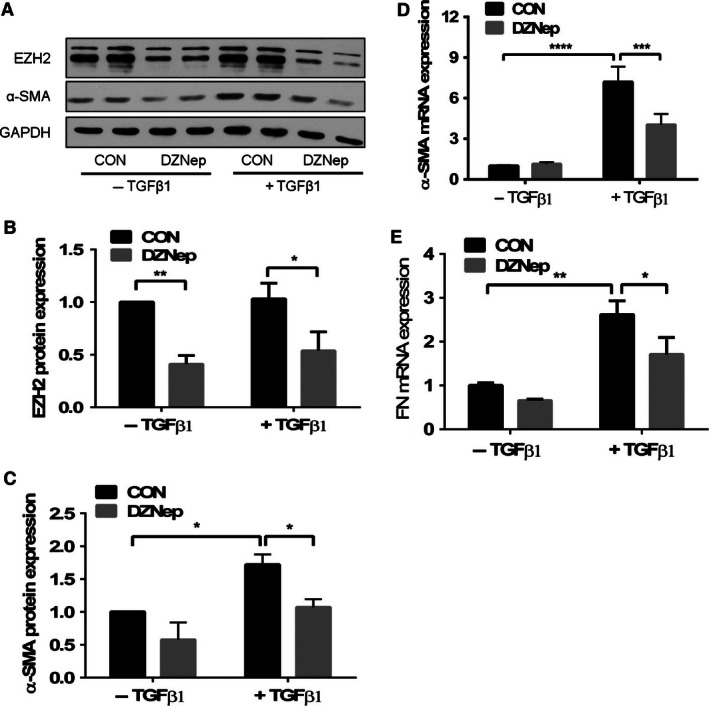
The TGF‐*β*1‐induced expression of myofibroblast markers is inhibited by the EZH2 inhibitor DNZep. The LL29 cells were treated with DZNep (4 *μ*mol/L) for 24 h and then with both DZNep and TGF‐*β*1 (5 ng/mL) for additional 24 h. The cells without DNZep treatment were used as a control (CON). The EZH2 and *α*‐SMA protein levels were measured by western blotting. GAPDH was used as a loading control. Representative blots are shown in (A), and the levels were quantitated using the ImageJ software and normalized to the GAPDH levels; the results are shown in (B) and (C). The levels of the *α*‐SMA (D) and fibronectin (FVC). (E) mRNAs were determined using real‐time PCR and normalized to the *β*‐actin levels. All results are expressed as the mean ± SE from three independent experiments, each performed in duplicate. *P < 0.05, **P < 0.01, ***P < 0.001, ****P < 0.0001 (ANOVA and Fisher's LSD).

To determine the function of EZH2 in IPF, we examined the effect of EZH2 inhibition on the differentiation of fibroblasts to myofibroblasts. We used the EZH2 inhibitor 3‐deazaneplanocin A (DZNep) for this experiment (Fiskus et al. 2009; Fujiwara et al. 2014). Human lung fibroblasts (LL29) were treated with DZNep and then stimulated with TGF‐*β*1. The western blot analysis showed that TGF‐*β*1 did not affect the expression of the EZH2 protein. However, DZNep reduced the EZH2 protein levels in both basal and TGF‐*β*1‐stimulated cells (Fig. 2A and B). TGF‐*β*1 increased the expression of the *α*‐SMA mRNA and protein, and the reduction of the EZH2 levels by DZNep inhibited the TGF‐*β*1‐induced *α*‐SMA expression (Fig. 2A, C and D). Furthermore, the mRNA level of fibronectin (FN), the marker of fibroblast activation, was also induced by TGF‐*β*1, but was inhibited by DZNep (Fig. 2E).

**Figure 3:**
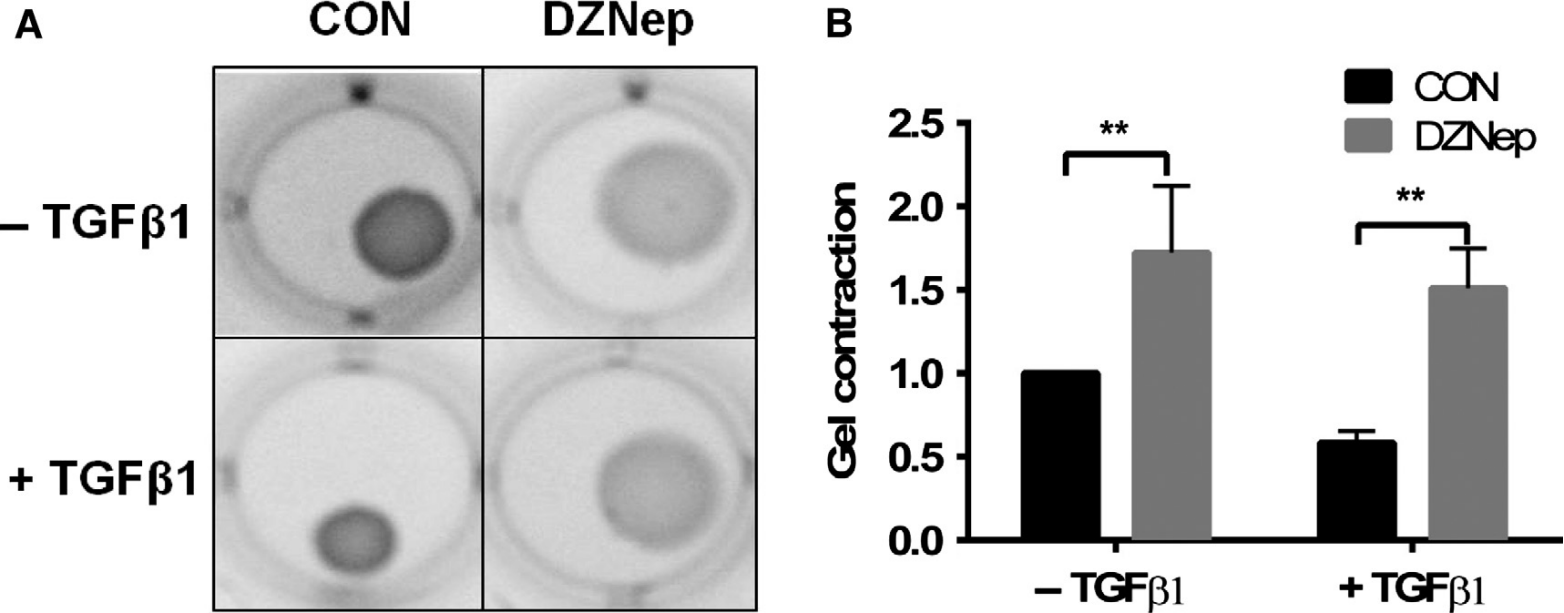
TGF‐*β*1‐induced contractility is inhibited by the EZH2 inhibitor DZNep. (A) The LL29 cells were treated with DZNep (4 *μ*mol/L) for 24 h and then with both DZNep and TGF‐*β*1 (5 ng/mL) for additional 24 h. The cells were mixed with collagen 1 and added to 12‐well plates. The gel was detached from well wall and bottom and the images were captured after 48 h. (A) Representative images. (B) The gel surface area was measured using ImageJ software. The results are shown as the mean ± SE from three independent experiments (*n* = 3); each experiment was performed in duplicate. **P < 0.01 (ANOVA and Fisher's LSD).

Contractility is one of the characteristics of fibroblasts/myofibroblasts and myofibroblasts have a stronger contractility than fibroblasts. We measured contractility using a gel contraction assay (Montesano and Orci 1988). The LL29 cells were pretreated with DZNep, followed by TGF‐*β*1 stimulation. The cells were trypsinized and mixed with collagen. After polymerization, the gels were detached from the bottom of the wells. Cell contractility causes the gel to shrink (Fig. 3A), and thus the surface area of the gel reflects the cellular contractility. The TGF‐*β*1‐treated cells had a smaller surface area compared to those without TGF‐*β*1 treatment. The inhibition of EZH2 by DZNep significantly increased the gel surface area in both the basal and TGF‐*β*1‐stimulated cells (Fig. 3B), indicating that DZNep reduced fibroblast/myofibroblast contractility.

**Figure 4:**
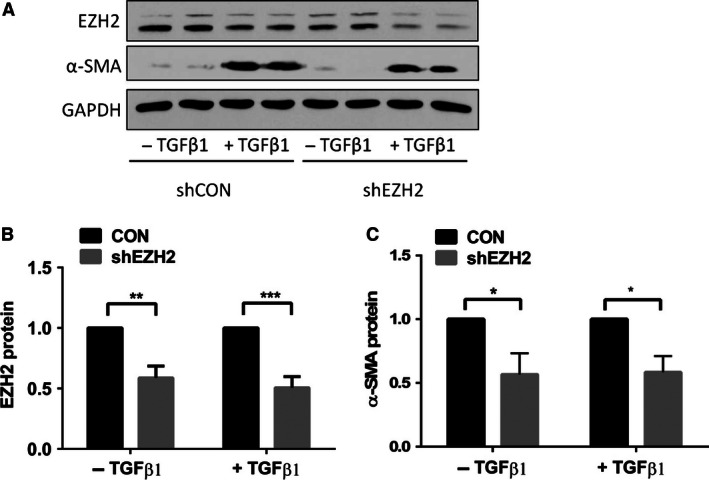
EZH2 knockdown decreases the expression of the myofibroblast marker *α*‐SMA. The LL29 cells were infected with a lentivirus containing shEZH2 or its control shCON at a MOI of 100 for 24 h. 48 h after infection, the cells were treated with 5 ng/mL of TGF‐*β*1 for an additional 24 h. The levels of the EZH2 and *α*‐SMA proteins were determined by western blotting. (A) Representative blots. (B, C) Quantitation of the western blotting results using ImageJ software. The results were normalized to GAPDH and expressed as a percent of shCON. The data are shown as the mean ± SE from three independent experiments, each performed in duplicate. *P < 0.05, **P < 0.01, ***P < 0.001 (ANOVA and Fisher's LSD).

To further confirm the inhibitor study, we reduced the EZH2 protein level using gene silencing. The LL29 cells were infected with an EZH2 shRNA or a control lentivirus at a MOI of 50 and then the cells were stimulated with TGF‐*β*1. The western blot analysis showed a reduction in EZH2 and *α*‐SMA protein levels in the EZH2 shRNA‐treated cells (Fig. 4). Taken together, our results suggest that the reduced EZH2 levels inhibit the differentiation of fibroblasts to myofibroblasts.

### EZH2 inhibition reduces p‐Smad2/3 nuclear translocation

**Figure 5:**
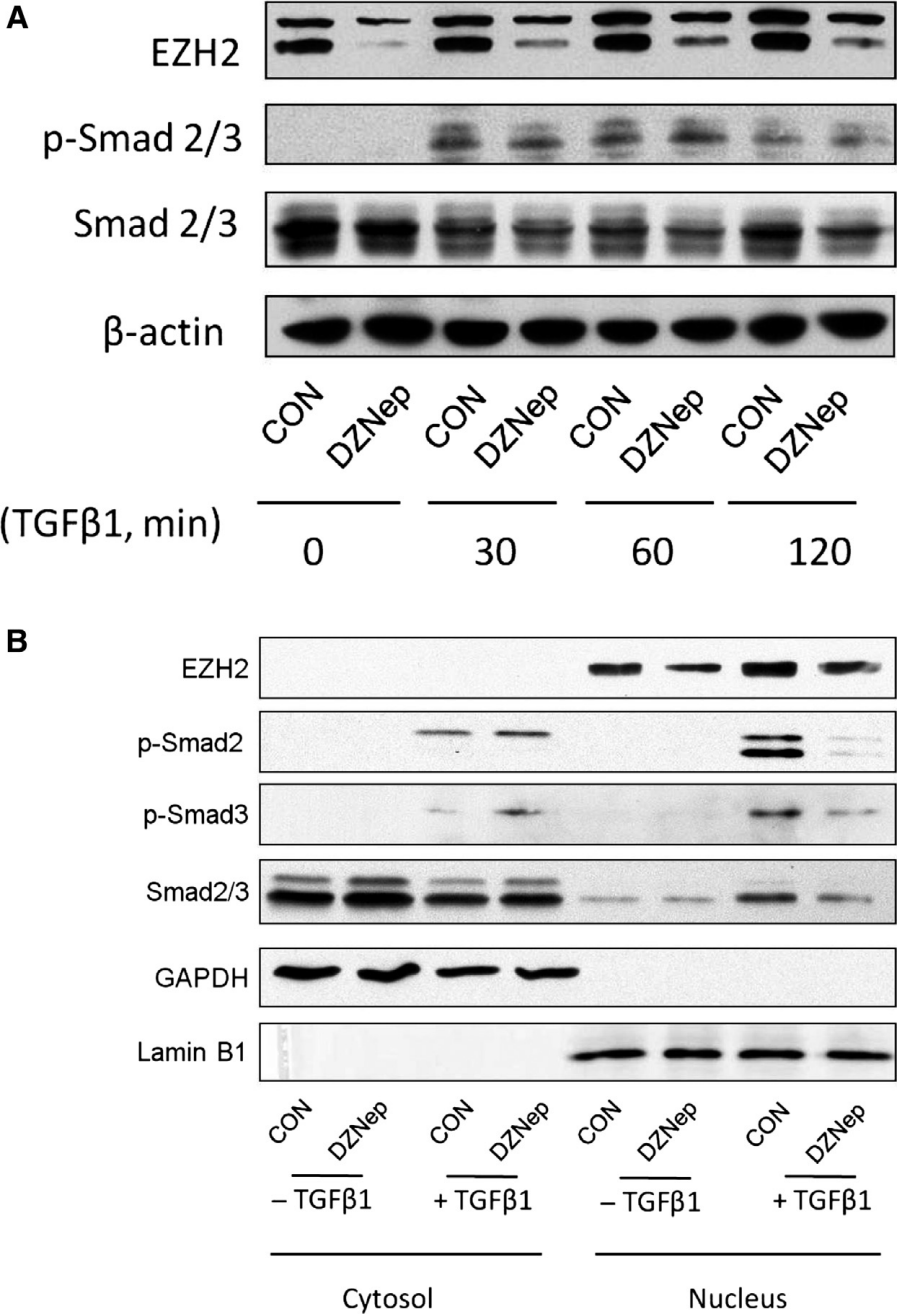
DZNep does not affect Smad2/3 phosphorylation but inhibits Smad2/3 nuclear translocation. (A) The LL29 cells were treated with 4 *μ*mol/L of DZNep for 48 h and then with TGF‐*β*1 (5 ng/mL) for 30, 60, or 120 min. Western blotting was performed to detect the levels of the EZH2, p‐Smad2/3, Smad 2/3, and *β*‐actin proteins. Representative results from three independent experiments are shown. (B) The LL29 cells were treated with DZNep (4 *μ*mol/L) or vehicle (CON) for 48 h, and then with TGF‐ *β*1 (5 ng/mL) for 60 min. The levels of the EZH2, p‐Smad 2, p‐Smad3, and Smad 2/3 proteins in the cytoplasmic and nuclear fractions were determined by western blotting. GAPDH and lamin B1 were used as loading controls for the cytosol and nucleus, respectively. Representative results from three independent experiments are shown.

TGF‐*β* signaling is initiated by the binding of TGF‐*β* to a complex of transmembrane receptor serine/threonine kinases (TβRI and II) on the cell membrane. The activated TβRI phosphorylates Smad2/3, which form a complex with Smad4. The Smad complex then translocates into the nucleus and regulates the transcription of downstream target genes. To explore how EZH2 affects TGF‐*β* signaling, we first determined whether DZNep affects the phosphorylation of Smad2/3. The LL29 cells were treated with DZNep for 48 h and then stimulated with TGF‐*β*1 for 30, 60, and 120 min. The TGF‐*β* treatment resulted in the phosphorylation of Smad2/3, as determined by western blotting using antibodies against phosphorylated Smad2/3. However, DZNep had little effect on the phosphorylation of Smad2/3 (Fig. 5A).

We next determined whether DZNep affected p‐Smad2/3 nuclear translocation. The LL29 cells were treated with DZNep for 48 h and stimulated with TGF‐*β*1 for 60 min. The nucleus was separated from the cytosol. The exclusive localization of cytoplasmic marker GAPDH in the cytosol and the nuclear marker lamin B1 in the nucleus confirmed the separation of the nucleus and cytosol (Fig. 5B). EZH2 was only located in the nucleus, and the level of its protein was reduced by the DZNep treatment. p‐Smad2 and p‐Smad3 were only detected in the TGF‐*β*1‐treated group, in which DZNep decreased the levels of p‐Smad2/3 in the nucleus, but increased them in the cytosol. This result indicates that the inhibition of EZH2 reduces p‐Smad2/3 nuclear translocation.

### DZNep attenuates the severity of bleomycin‐induced lung fibrosis

**Figure 6:**
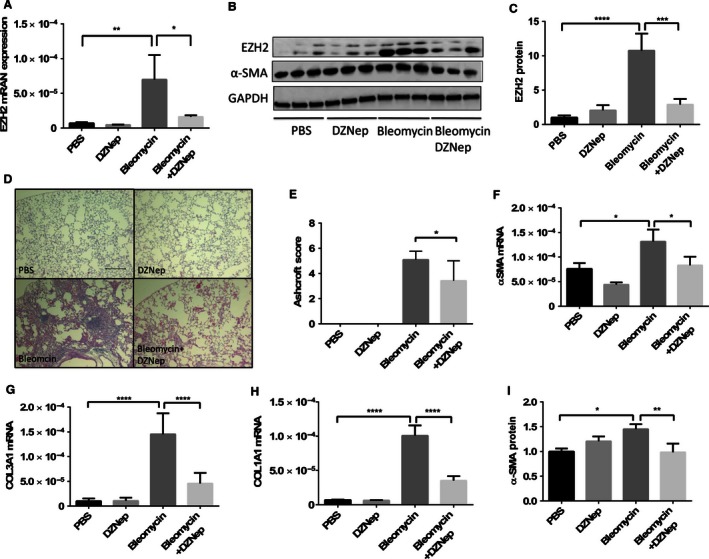
DZNep attenuates pulmonary fibrosis. Female C57BL/6 mice were intratracheally challenged with PBS or bleomycin and treated with PBS or DZNep through intraperitoneal injection once per day starting 1 day before the bleomycin challenge. On day 17, the left lung tissue was collected for RNA and protein extraction and the right lung tissue was fixed for hematoxylin and eosin staining. (A) The levels of the EZH2 mRNA were determined using real‐time PCR and normalized to the 18S rRNA. The levels of the EZH2 protein were determined by western blotting. Representative blots and quantitation are shown in (B, C). The results were normalized to GAPDH. The data are shown as the mean ± SE. *n* = 6 animals in each group. *P < 0.05, **P < 0.01, ***P < 0.001, ****P < 0.0001 (ANOVA and Fisher's LSD). (D) Representative images of the H & E staining. Scale bar: 200 *μ*m. (E) The Ashcroft scale was used to quantify the pulmonary fibrosis. All the results are expressed as the mean ± SE. *n* = 6 animals in each group. *P < 0.05 (Student's *t*‐test). The mRNA levels of the fibrotic markers *α*‐SMA (F), COL1A1 (G), and COL3A1 (H) were determined using real‐time PCR and normalized to the 18S rRNA. The levels of the *α*‐SMA protein were determined using western blotting. Representative blots and quantitation are shown in (B, F). The data were normalized to GAPDH. All results are shown as the mean ± SE. *n* = 6 animals in each group. *P < 0.05, **P < 0.01, ****P < 0.0001 (ANOVA and Fisher's LSD). PBS, phosphate‐buffered saline.

To determine the in vivo effect of EZH2 inhibition on lung fibrosis, we intraperitoneally administered DZNep to the mice daily beginning 1 day before the bleomycin challenge. The lung tissues were collected on day 17. Bleomycin increased the expression of the EZH2 mRNA by 10‐fold compared to the PBS group (6.96 ± 1.46×10^−6^ vs. 6.98 ± 3.54×10^−5^), and the DZNep treatment attenuated the increase by 77% (Fig. 6A). Similar changes were observed for the EZH2 protein, with a 10.8‐fold increase in the bleomycin group compared to control group and 73% inhibition by DZNep (Fig. 6B and C). This result confirmed the effectiveness of the DZNep treatment in reducing the EZH2 expression levels in vivo.

The H&E staining showed that while the lung tissue of the mice was normal in both the PBS‐ and DZNep‐treated groups, the lung tissue of the bleomycin‐treated mice exhibited thickened alveolar septa and fibroblasts/myofibroblasts foci. The lungs of the bleomycin plus DZNep group exhibited a marked reduction in the fibrotic areas (Fig. 6D). The severity of fibrosis then was evaluated using a semiquantitative histological scoring system that was introduced by Ashcroft et al. (1988). A higher Ashcroft score represents more severe fibrosis, as measured by histology. The Ashcroft scores in the PBS and DZNep groups were zero. In the bleomycin‐challenged group, the Ashcroft score was 5.09 ± 0.27, which was reduced to 3.43 ± 0.64 by the DZNep treatment (Fig. 6E).

To further assess the severity of the lung fibrosis in each group, we measured the mRNA and/or protein expression of myofibroblast markers. As expected, the mRNA level of *α*‐SMA was upregulated by 73% following the bleomycin challenge (7.61 ± 1.62 × 10^−5^ vs. 13.2 ± 0.2 × 10^−5^), and this increase was decreased to 37% by the DZNep treatment (Fig. 6F). Similar changes were observed for the expression of the COL1A1 and COL3A1 mRNAs (Fig. 6G and H). The *α*‐SMA protein was also upregulated in the bleomycin‐treated group and downregulated by the DZNep treatment (Fig. 6I). All the results suggest that DZNep reduces the severity of bleomycin‐induced fibrosis in the lungs.

## Discussion

Previous studies on EZH2 have mainly focused on its histone methyltransferase activity (Chase and Cross 2011; Fiskus et al. 2009; Fujiwara et al. 2014; Hussain et al. 2009; Kikuchi et al. 2012; Simon and Lange 2008; Yang and Yu 2013). The purpose of this study was to examine the role of EZH2 in the development of IPF. We found that EZH2 was upregulated in the lungs of patients with IPF and in bleomycin‐challenged mice. The inhibition of EZH2 by the inhibitor DZNep or an EZH2 shRNA reduced fibroblast activation in vitro and in vivo. EZH2 mediates this effect by enhancing the TGF‐*β* signaling pathway, likely through pSmad2/3 nuclear translocation.

IPF and cancer share some common characteristics (Antoniou et al. 2015; Vancheri 2013; Vancheri et al. 2010). Several signaling pathways, such as the Wnt/β‐catenin signaling pathway, are abnormally activated in both IPF and cancer (Chilosi et al. 2003; Mazieres et al. 2005). The global DNA methylation patterns observed in patients with IPF have some similarities to those in cancer patients (Rabinovich et al. 2012; Sanders et al. 2012). Approximately 10% of the microRNAs are significantly changed in IPF, and some of them are involved in this disease (Cao et al. 2010; Cushing et al. 2011; Lovat et al. 2011; Pandit et al. 2010, 2011). Many microRNAs also contribute the pathogenesis of cancer (Lovat et al. 2011; Oak et al. 2011). By taking advantage of the findings from cancer patients, we may be able to better understand the pathogenesis of IPF (Vancheri 2013).

The downregulation of miR‐101 in cancer leads to the overexpression of its target, EZH2, which results in cancer progression (Cao et al. 2010; Varambally et al. 2008). The overexpression of EZH2 is associated with prostate cancer, breast cancer, bladder cancer, and lung cancer (Simon and Lange 2008; Yang and Yu 2013). Although EZH2 has extensively been studied in several human cancers, its role in IPF is still unknown. Similar to cancer, we found that EZH2 was upregulated in IPF and its expression level was inversely correlated to the pulmonary functions of patients with IPF. EZH2 was colocalized with the myofibroblast marker *α*‐SMA in the bleomycin‐challenged mice. This result was confirmed by the observation that EZH2 expression was higher in the primary fibroblasts/myofibroblasts isolated from the bleomycin‐challenged mice than those from the control mice. The upregulation of EZH2 in the *α*‐SMA‐expressing myofibroblasts suggests that EZH2 may regulate the differentiation of fibroblasts to myofibroblasts.

The uncontrolled extracellular matrix production in IPF originates from fibroblastic foci (Enomoto et al. 2006; Flaherty et al. 2003; Hardie et al. 2009). Resident fibroblasts, epithelial cells (via EMT), and fibrocytes contribute to the formation of the foci (King et al. 2011). Three factors are known to induce the differentiation of fibroblasts to myofibroblasts: local accumulation of active TGF‐*β*1, high extracellular mechanical stress, and the presence of specialized ECM proteins (Hinz et al. 2007). The current study also showed that TGF‐*β*1‐treated human fibroblasts exhibited increased expression of *α*‐SMA and ECM proteins, such as Fn, as well as collagen gel contractility. The EZH2 inhibitor DZNep reduced the TGF‐*β*‐induced expression of these proteins and gel contractility. Furthermore, the reduction in the EZH2 level by an EZH2 shRNA also reduced the levels of the *α*‐SMA protein in the TGF*β*1‐treated fibroblasts. These data suggest that the inhibition of EZH2 by DZNep prevents the differentiation of fibroblasts to myofibroblasts.

The TGF‐*β* signaling pathway is involved in *α*‐SMA expression and myofibroblast differentiation (Adam et al. 2000; Grotendorst et al. 1996). Upon phosphorylation, Smad3 binds to the Smad3‐binding elements (SBEs) in the *α*‐SMA promoter to induce *α*‐SMA expression in myofibroblasts (Hu et al. 2003). Smad3 is also required for ECM secretion in myofibroblasts (Schnabl et al. 2001). The TGF‐*β* signaling pathway is regulated by many mechanisms (Derynck and Zhang 2003). Increased phosphorylation of Smad2/3 activates TGF‐*β* signaling. However, the inhibition of EZH2 using DZNep had no effect on the levels of total or phosphorylated Smad2/3. Thus, this is not likely the mechanism of EZH2‐induced myofibroblast differentiation.

The dynamic distribution of Smads between the cytoplasm and nucleus tightly regulates the TGF‐*β* signaling pathway. The translocation of phosphorylated Smads from the cytoplasm into the nucleus is considered as a rate‐limiting step in TGF‐*β* signal transduction. The export of Smads out of the nucleus to the cytoplasm turns off the signaling. The nuclear import factors moleskin (Msk), importin 7 (Imp7), and Imp8 are responsible for Smad nuclear import (Xu et al. 2007; Yao et al. 2008) by interacting with nucleoporins, including Sec13, Nup75, Nup93, and Nup205 (Chen and Xu 2010). Exportin 4 and RanBP3 have been reported to be involved in Smad nuclear export (Dai et al. 2009; Kurisaki et al. 2006). Our result indicates that the inhibition of EZH2 reduced Smad2/3 accumulation in the nucleus. However, the mechanism remains to be determined. Also, we cannot rule out the possibility that EZH2 exerts its effect via histone methyltransferase activity (Chase and Cross 2011; Fiskus et al. 2009; Fujiwara et al. 2014; Hussain et al. 2009; Kikuchi et al. 2012; Simon and Lange 2008; Yang and Yu 2013).

Several mechanisms regulate Smad trafficking between the cytoplasm and nucleus (Hill 2009). Monoubiquitination of Smad4 by the ubiquitin ligase TIF1*γ* reduced the accumulation of Smad4 (Dupont et al. 2009). Phosphorylation in the linker region by cyclin‐dependent kinases (CDKs) and MAPK inhibits Smad translocation (Kretzschmar et al. 1997; Sapkota et al. 2007). A kinetic analysis of Smad nucleocytoplasmic trafficking shows that the nuclear mobility of Smad2 and Smad4 and the nuclear export rate of Smad2 are decreased in TGF‐*β*1‐treated cells, indicating that the active Smads are trapped in the nucleus (Schmierer and Hill 2005). Smad trapping can be explained by the interaction of Smads with other proteins in the nucleus, which retains the Smads in the nucleus. The transcription factor Yes‐associated protein (YAP) and transcriptional coactivator with PDZ‐binding motif (TAZ) have been shown to directly bind Smads to retain the Smads in the nucleus (Varelas et al. 2008, 2010).

EZH2 can interact with several proteins. EZH2 binds EED, SUZ12, and other proteins to form PRC2, which catalyzed H3K27me3; it also interacts with transcription factor YY1 (Caretti et al. 2004). A recent study demonstrated that EZH2 was able to directly bind Smad2 and Smad4 (Wang et al. 2013). However, this experiment was done by cotransfection of EZH2 and Smad plasmids, which is not physiological relevant. We could not confirm this interaction endogenously by coimmunoprecipitation of fibroblast cell lysate (data not shown).

Several animal models of experimental lung fibrosis have been developed for studying the pathogenesis of IPF, including exposure to bleomycin, silica, fluorescein isothiocyanate, irradiation, or the expression of specific genes using a transgenic system (Degryse and Lawson 2011). Bleomycin is an anticancer chemotherapeutic drug that causes pulmonary fibrosis as a major adverse effect. It directly breaks the DNA strand and induces oxidative stress to cause fibrosis (Moeller et al. 2008). Although the bleomycin model has some limitations (Degryse and Lawson 2011; Moeller et al. 2008), a single intratracheal dose of bleomycin remains the mostly frequently used method, mainly due to its easy delivery, short time to induce fibrosis, and some histological hallmarks that are similar to IPF.

Using intratracheal delivery of a single dose of bleomycin (DuPage et al. 2009), we found that EZH2 was significantly upregulated. This model provides us a tool to evaluate the effect of the EZH2 inhibitor DZNep on pulmonary fibrosis in vivo. DZNep does not have any obvious toxicity when administered in a single dose of 10 mg/kg (Coulombe et al. 1995). The effects of DZNep in various cancer models have been reported. An intravenous administration of DZNep inhibits angiogenesis in a subcutaneous glioblastoma mouse model, without toxicity (Smits et al. 2011). The intraperitoneal delivery of DZNep and the histone deacetylase inhibitor panobinostat increases animal survival in an acute myeloid leukemia mouse model (Fiskus et al. 2012). In the leukemia‐engrafted mouse model of AML, the intraperitoneal administration of DZNep reduces tumor growth and prolongs the animals’ survival (Zhou et al. 2011). DZNep‐treated prostate cancer cells exhibited reduced tumorigenicity in vivo (Crea et al. 2011). In this study, we found that an intraperitoneal administration of DZNep reduced pulmonary fibrosis in the bleomycin‐induced pulmonary fibrosis mouse model.

In summary, we discovered that EZH2 is upregulated in IPF and it promotes the differentiation of pulmonary fibroblasts to myofibroblasts. We also revealed a new mechanism of EZH2 action, that is, enhancing the TGF‐*β* signaling pathway by promoting Smad2/3 nuclear translocation. In vivo studies showed that the inhibition of EZH2 by DZNep reduced pulmonary fibrosis.

## Conflict of Interest

None declared.
